# Comparison of Chromium and Iron Distribution in Serum and Urine among Healthy People and Prediabetes and Diabetes Patients

**DOI:** 10.1155/2019/3801639

**Published:** 2019-02-24

**Authors:** Qi Zhou, Wenjia Guo, Yanan Jia, Jiancheng Xu

**Affiliations:** ^1^Department of Pediatrics, First Hospital of Jilin University, Changchun 130021, China; ^2^Department of Laboratory Medicine, First Hospital of Jilin University, Changchun 130021, China

## Abstract

The effect of chromium (Cr) and iron (Fe) on prevalence of diabetes has received great attention. This study investigated serum and urinary Cr and Fe levels among patients with impaired fasting glucose (IFG), impaired glucose tolerance (IGT), type 1 diabetes (T1D), and type 2 diabetes (T2D) in the Northeast Chinese population. From January 2010 to October 2011, patients with IFG (*n*=12), IGT (*n*=15), T1D (*n*=25), T2D (*n*=137) and healthy controls (*n*=50) were enrolled in the First Hospital of Jilin University. Trace elements were detected using an inductively coupled plasma spectrometer. Serum Cr levels decreased in T2D without complications, diabetic retinopathy (DR), diabetic peripheral neuropathy (DPN), and diabetic nephropathy (DN) (*P*<0.05). The urinary Cr level in T1D was the highest of all, which significantly exceeded those of the T2D groups with and without complications. No significant differences of serum Fe levels were found among all groups. The urinary Fe level of T1D was significantly increased (*P*<0.05). The correlation between serum Cr and serum Fe in T2D was obviously positive (*P*<0.05). One month of simvastatin therapy exerted no effects on serum or urinary Cr and Fe levels. These results suggest the potential role of Cr and Fe in diabetes should receive attention.

## 1. Introduction

Incidence of diabetes mellitus (DM) has been remaining high worldwide. The chronic disease has caused 1.6 million people to die in 2015 [1] and WHO predicts the number of patients would be over 592 million people in 2035 [2]. As the 4th-fatal illness of the noncommunicable diseases [1], it worsens patients' health and quality of life severely. Previous studies have shown increasing prevalence of diabetes in China, which is the world's largest diabetes epidemic now.

Among the Chinese adult population in 2013, the estimated standardized prevalence of total diagnosed and undiagnosed diabetes is 10.9%; that of diagnosed diabetes, 4.0%; and that of prediabetes, 35.7% [3]. Diet plus physical activity may reduce the incidence of type 2 diabetes (T2D) in people with impaired glucose tolerance (IGT).

Basic and clinical studies reveal that transportation, distribution, excretion, and accumulation of various kinds of trace elements under diabetic condition changed differently [4–6] and influence development of diabetes and complication progression dissimilarly [7]. Considering the nosogenesis mechanism, chromium (Cr) and iron (Fe) promote diabetes through insulin resistance solely [8, 9]. Some scholars believe Cr benefits the human body [10]. Cr promotes glycolysis in muscle cells and fat cells, acts as an inhibitor of glycogen decomposition in myocytes, and is able to regulate glucose according to a variety of animal models and clinical trials. It is classified as a “hypoglycemic metal element” in the review of Adrian et al. [11], which is involved in carbohydrate, lipid, and protein metabolism primarily by increasing insulin efficiency [12]. Chromium-containing compounds enhance insulin activity, and what is approved is that insulin-responsiveness is improved in diabetic patients after supplementation with chromium-based compounds [13]. Its mechanism may be increasing the concentration of the insulin receptor RNA messenger; complex formation with insulin enhancing its activity; and increasing islet *β* cell sensitivity by reducing concentration of TNF-*α*, resistin, and interleukin-6 [14]. Many clinical studies have confirmed the decrease of serum Cr in patients with T1D and T2D [15, 16] with a drastic increase of Cr in urine [17]. Plasma Cr is negatively correlated with glucose in patients with T2D [18].

Fe is an essential element of organisms, a constituent of various proteins and enzymes. There has been no experiment so far proving that Fe directly increases glucose, while evidence shows it damages the pancreas and induces diabetes [11]. It is currently believed that Fe overload strongly associates with insulin resistance, hyperglycemia, and high risk of T2D [19]. For example, ferritin, the index for Fe storage, is correlated with diabetes [20]. Some researchers believe that Fe induces diabetes through oxidative stress and direct damage towards pancreatic *β* cells [21]. In addition, the other side is that frequent blood donation (≥2 times per year) is considered to be a protective factor for diabetes, which reduces Fe reserve, increases insulin sensitivity, and weakens postprandial hyperinsulinemia [22].

The function of each element is different and their relationships appear to be complex, as calcium, magnesium, titanium, zinc, vanadium, and Fe reduce Cr absorption [23]. Therefore many investigators now turn to “pack” them to analyze the changes and roles of several more related ones. We have done so as with magnesium and calcium, copper, and zinc [5, 6]. Because both Cr and Fe function as an insulin resistant, we conduct this comparative study on the distribution and correlation of Cr and Fe among healthy, prediabetic, and diabetic populations and try to explore the interaction of the two. By exploring their levels in the serum and urine of subjects, respectively, we also intend to track metabolic changes and disease-relevant information subsequently.

## 2. Materials and Methods

### 2.1. Ethical Statements

This retrospective study was approved by the Ethics Committee of the First Hospital of Jilin University. All patients provided signed informed consent. Data were obtained from electronic medical records of the hospital, and the information was anonymous.

### 2.2. Subjects

To ensure academic integrity and rigor, subject selection of this study is the same as those of our previous researches [5, 6], which can be described briefly as 189 definitively diagnosed patients enrolled from January 2010 to October 2011 in the First Hospital of Jilin University and grouped based on medical certification as follows: impaired fasting glucose (IFG,* n*=12 people), impaired glucose tolerance (IGT,* n*=15), type 1 diabetes (T1D,* n*=25), type 2 diabetes without complications (T2D,* n*=29), diabetic nephropathy (DN,* n*=24), diabetic retinopathy (DR,* n*=34), and diabetic peripheral neuropathy (DPN,* n*=50). The control group (CON,* n*=50) was enrolled according to the age and sex proportion from physical examination group at the same standard meantime, noting that subjects had not received any element supplement due to official approval of effectiveness. The subjects' basic information as age, sex, and BMI will not be detailed here to avoid redundancy and duplication [5].

### 2.3. Element Measurement

The elements' measurements were the same as previous studies of our research project [5, 6], using ICP-MS. The quality control of all analyzed samples was performed by using standard reference materials from the China Standard Material Center. Limits of detection (LOD) were 1.0 *μ*g/L for Cr and Fe. The recovery of standard trace elements (accuracy) ranged from 93.3% to 98.9%.

### 2.4. Statistics

Statistical description was performed as median (interquartile range); the Kruskal-Wallis test was used for evaluation among multigroups, and the comparison between groups was assessed applying the Mann-Whitney test with correction by the Bonferroni method of *α*; correlation was analyzed with Spearman's method. The* P *value reported was two-sided and statistically significant at* P *< 0.05. All analysis was conducted using SPSS 24.0.

## 3. Results and Discussion

### 3.1. Trace Elements Levels in Prediabetes and Diabetes

**Figure 1:**
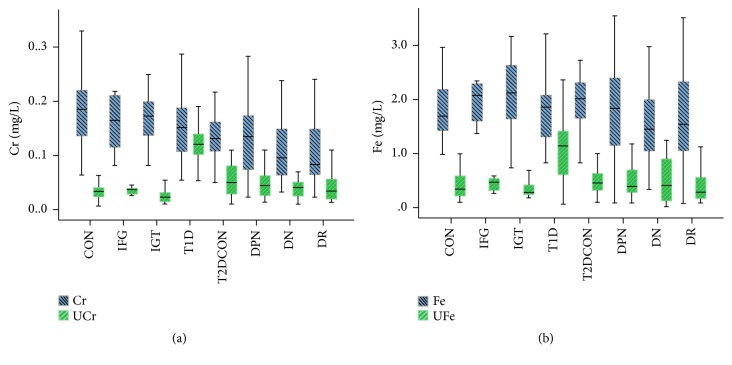
Levels of Cr and Fe in the healthy control, IFG, IGT, T1D, T2D without complications, DPN, DN, and DR groups. Boxplots display the extreme, the upper and lower quartiles, and the median of the maximum difference in the healthy control, IFG, IGT, T1D, T2D without complications, DPN, DN, and DR groups. The median for each dataset is indicated by the centerline, and the first and third quartiles are presented by the edges of the area, which is known as the interquartile range (IQR). (a) Serum Cr levels decreased in the T2DCON, DR, DPN, and DN group (*P*<0.05) and urinary Cr level in T1D was the highest; (b) urinary Fe level of T1D was significantly increased (*P*<0.05).

Serum glucose and HbA1c were tested to support the enrollment standard.  Table 1 showed that, compared with the control group, serum Cr levels decreased in the T2DCON, DR, DPN, and DN groups (*P*<0.05); though no statistical significance (*P*>0.05) was shown, a downward trend appeared in IFG, IGT, and T1D. On the other hand, the urinary Cr level in T1D was the highest of all (Figure 1(a)), which significantly exceeded those of the T2D groups with and without complications. Surprisingly, we barely found significant difference of serum Fe levels among all groups. The urinary Fe level of T1D was significantly increased (*P*<0.05) and reached the peak (Figure 1(b)), while that of T2D was lower (*P*<0.05).

### 3.2. Correlation Analysis of Trace Elements

As presented in Table 2, the correlation between serum Cr and serum Fe in T2D was positive obviously (*P*<0.05); meanwhile these relations in IFG, IGT, and T1D were not statistically significant (*P*>0.05).

### 3.3. Effect of Simvastatin Treatment on Trace Elements in T2D Patients

Statin treatment was often used to lower lipid in the patients with T2D. Therefore the effect of simvastatin therapy for one month on serum or urinary Cr and Fe levels was detected in the patients with T2D. As presented in Table 3, one month of simvastatin therapy had no effects on serum or urinary Cr and Fe levels.

## 4. Discussion

### 4.1. Comparison of Serum and Urinary Cr

It is currently believed that diabetic patients are under a state of trace elements disorder, such as insulin promoting discrepancy of distribution of multiple ions in the bloodstream [4]. More than 80% of the Cr in the human body is excreted in urine [24], and the amount of urinary Cr in T1D patients is considered to be more than twice that of the control group [17]. Some scientists attributed this to its increased loss plus decreased absorption [25]. Therefore, we believe the simultaneous study of both serum and urine trace element levels may provide better overview of the state and degree. We confirmed serum Cr levels were lower in the T2DCON, DR, DPN, and DN population than that of the control, which is consistent with findings of other investigators [7, 26, 27]; IFG and IGT had lower serum Cr levels than the control even with no statistical significance, which is similar to the conclusion of Chen [27]; the comparison of serum Cr between T1D and the control showed no significant difference and was consistent with Lin's study [28] of T1D children, adults, and healthy people.

As for urinary Cr, the sum that T2D patients have higher levels of urinary Cr was the same as the proposition of Kazi et al. [7] who proved no statistically significant difference as well but deemed the tendency existed; it elevated visibly in T1D; considering the severity of illness and thus selection being incomprehensive, prediabetes showed no significant difference comparing with the control group on urinary Cr level; the Cr/UCr in diabetic groups was lower than those of the prediabetic and control group, indicating diabetic patients were all under low Cr state. It is already proved in a research launching in Indian rural areas that elevated urinary Cr is strongly correlated to diabetes [29]; our experiment adopted a random urine specimen, which might have varied greatly due to physical and chemical factors; thus the statistical difference could not be that obvious. Besides, it is noticeable that the excretion rate of Cr and Fe changed mildly in DN patients, which was opposite to our expectation that severe renal function damaged influenced elements in urine.

Combined with serum Cr information, it was stated in our experiment that T2D patients lied in low Cr condition, the same as findings of relatively low Cr content in T2D patients' hair [7] and nails [30]. That explained what Cefalu WT [31] purposed in which low Cr induced upward fasting glucose and glycosuria. T1D patients exhibited a negative Cr balance [32], which was in consistency with the results of no significant change of serum Cr and overt increase of urinary Cr in our study. It is stated that the T1D metabolic control system required additional Cr whereas the absorbed element was not utilized and discharged in urine [17].

Besides, Cr/UCr results also revealed that diabetic patients were losing Cr. Morris [18] found diabetic patients had a 33% reduction of plasma Cr and 100% increase of urinary Cr. Our experiment could not find stationary correlation between Cr and lipid indicators which was testified in a lot of animal and clinical studies [4, 31, 33, 34], possibly resulting from therapeutic regimens and medications.

As an enhancer for insulin function, Cr participates in carbohydrate, lipid, and protein metabolism by improving insulin efficiency, and its deficiency seriously affects glucose tolerance and lipid homeostasis [12]. The concrete mechanism remains unknown [12], whereas studies confirmed Cr regulated glucose in a variety of pathways. For example, Cr is involved in hyperinsulinemia and insulin resistance [35]; Vincent et al. [36] demonstrated that chromodulin was able to activate insulin receptor kinase; Pattar et al. [37] believed Cr altered membrane fluidity and regulated glucose uptake consequently by changing cholesterol content; it might impact structure of lipid bilayer [38] and the like. Both animal experiments [39] and clinical studies [40–42] have affirmed that Cr supplementation reduced glucose; therefore experts suggested [43] that that be applied as T2D complementary therapy.

However, healthy people did not benefit from that tentative plan [44]. Therefore, Lewicki S [12] thought this treatment was probably only valid to a population with metabolic disorders, which should be supplemented as appropriate.

### 4.2. Comparison of Serum and Urinary Fe

Surprisingly, there was no significant difference of serum Fe levels among the groups in our study, which was contrary with others' [45, 46]. Most current views were that diabetic patients are being Fe overloaded; evidence stood as diabetes associating with elevated ferritin [47]. Yet some data turned out that serum Fe level was not distinctive between diabetic patients and healthy people [15, 47–49]. Duan et al. [47] considered hyperglycemia led to enhancing oxidative stress that reduced free iron in circulation, with elevated hepcidin inhibiting the intestine absorbing Fe and release of Fe by the reticuloendothelial system; the correlation between serum Cr and serum Fe in IFG and IGT was not significant, but we still found the tendency existed. Therefore it might be ascribed to small sample size or case selection. Urinary Fe in T1D increased significantly and was much higher than that of T2D and the control. Additionally, Fe/UFe in T1D was apparently lower than other groups', indicating more Fe excretion. In the present study, serum Fe of DPN was not very disparate from that of CON and DN; the DPN and DR group encountered the same situation, which might result in no significant discrepancy. In the present study, one month of simvastatin therapy had no effects on serum or urinary Fe levels.

### 4.3. Correlation of Cr and Fe

Because of wide variety and complex mechanism of trace elements in the body, their mutual correlation and influence are still inconclusive in this emerging field. We have observed a significant correlation between serum Cr and serum Fe, in accordance with previous findings from supplement experiments [50, 51], possibly due to the cotransport mechanism of Cr and Fe resulting competition for binding sites of transferrin.* In vivo *and* in vitro* studies in rats have shown that approximately 80% of the Cr in the blood is associated with transferrin [52]. After being absorbed from the intestinal tract, Cr combines with proteins related to Fe metabolism, forming a complex transported into cells. The efficiency of the compound passing through the membrane depends on insulin concentration [53]. The bioavailability of Fe in rats treated with Cr intraperitoneally was reduced, and the animal even developed symptoms of anemia [54].

Our experiment concluded that patients with T2D were in low Cr state. The levels of urinary Cr and Fe in T1D patients were higher than those of T2D patients. It is suspected that loss and degree of disorder of trace elements in T1D patients are more severe. We assumed that diabetes affected absorption, transportation, and utilization of Cr and Fe. The limitations of this experiment are that the number of enrolled cases was not enough and the prediabetic group consisted of outpatients; therefore it would be difficult to follow up; there is no more detailed research, such as disease-staging patients; in a way, a 24 h urine sample may be better than a random urine specimen, which reflects cumulative exposure, exposure approaches, and different forms of elements [29]; metabolism, lifestyle, or drugs may not be excluded from impact of exposure, absorption, or excretion of certain elements.

We have not found any significant difference after simvastatin treatment. However, the data presented that serum Cr elevated and serum Fe and urinary Cr and Fe decreased after treatment. The efficacy might be obvious and statistically significant if the duration was set longer.

## 5. Conclusions

Trace elements and diabetes affect each other mutually. Research is now clinically focused on supplement effectiveness [55, 56] and control glucose as a result [47, 48]. For the reason that it has not yet accessibly arrived at an evidence-based aspect, there is no final conclusion concerning safety and availability [56]. What remains a current and difficult point in future research is to accurately extract and analyze the relationship and mechanism of synergy and antagonism through ingenious experimental design and research.

## Figures and Tables

**Table 1:** Comparison of indexes among groups [*M*(*P*_25_~ *P*_75_)].

	CON	IFG	IGT	T1D	T2DCON	DN	DR	DPN
	(*n*=50)	(*n*=12)	(*n*=15)	(*n*=25)	(*n*=29)	(*n*=24)	(*n*=34)	(*n*=50)
Glu (mmol/L)	4.8	6.4^1^	5.9^1,2^	11.9^1,2,3^	9.8^1,2,3^	8.2^1,2,3^	9.2^1,2,3^	8.$8^{\begin{array}{c} 1,2,3 \end{array}}$
	(4.1-5.6)	(6.3-6.7)	(5.6-6.1)	(8.5-14.8)	(8.3-12.8)	(7.5-9.5)	(7.7-11.4)	(7.6-11.1)
HbA1c (%)	5.1	6.1^1^	5.5^2^	11.3^1,2,3^	8.5^1,2,3,4^	8.5^1,2,3,4^	8.6^1,2,3,4^	8.$2^{\begin{array}{c} 1,2,3,4 \end{array}}$
	(4.2-6.0)	(5.9-6.2)	(5.4-5.7)	(10.3-12.8)	(7.7-8.9)	(8.0-8.8)	(8.0-8.9)	(7.2-8.7)
Cr (mg/L)	0.185	0.164	0.172	0.151	0.131^1^	0.096^1^	0.083^1,3,4^	0.13$5^{\begin{array}{c} 1 \end{array}}$
	(0.136-0.221)	(0.098-0.214)	(0.128-0.202)	(0.104-0.191)	(0.107-0.168)	(0.062-0.149)	(0.064-0.149)	(0.073-0.173)
UCr (mg/L)	0.034	0.038	0.023	0.120^1,2,3^	0.050^1,3,4^	0.040^4^	0.035^4^	0.04$4^{\begin{array}{c} 1,3,4 \end{array}}$
	(0.024-0.041)	(0.027-0.039)	(0.015-0.034)	(0.094-0.141)	(0.028-0.082)	(0.023-0.050)	(0.020-0.057)	(0.026-0.063)
Cr/UCr	5.329	5.511	7.353	1.270^1,2,3^	2.750^1,3,4^	2.525^1,3,4^	3.232^1,3,4^	3.16$8^{\begin{array}{c} 1,3,4 \end{array}}$
	(3.810-8.852)	(3.292-6.165)	(5.577-10.227)	(0.980-1.576)	(1.415-4.800)	(1.445-5.265)	(1.386-4.636)	(1.973-4.300)
Fe (mg/L)	1.697	2.087	2.116	1.857	2.014	1.447	1.539	1.841
	(1.402-2.185)	(1.554-2.303)	(1.454-2.650)	(1.172-2.341)	(1.587-2.410)	(1.027-2.025)	(1.025-2.350)	(1.147-2.409)
UFe (mg/L)	0.335	0.466	0.279	1.140^1,2,3^	0.450^4^	0.410^4^	0.290^4^	0.38$5^{\begin{array}{c} 4 \end{array}}$
	(0.199-0.585)	(0.301-0.538)	(0.216-0.425)	(0.595-1.465)	(0.310-0.700)	(0.108-0.908)	(0.159-0.553)	(0.279-0.803)
Fe/UFe	4.784	4.382	7.343	1.803^1,2,3^	3.744^4^	5.077	6.092	4.12$0^{\begin{array}{c} 4 \end{array}}$
	(3.023-7.879)	(3.394-6.389)	(3.421-10.727)	(1.061-3.445)	(2.498-6.997)	(1.662-10.395)	(1.595-9.896)	(2.679-7.522)

UCr refers to urinary Cr; UFe refers to urinary Fe. ^1^Compared with the CON group, there is significant difference. ^2^Compared with the IFG group, there is significant difference. ^3^Compared with the IGT group, there is significant difference. ^4^Compared with the T1D group, there is significant difference. ^5^Compared with the T2DCON group, there is significant difference. ^6^Compared with the DN group, there is significant difference.

**Table 2:** Correlation between Cr, Fe, and other indicators in serum and urine within each group.

		CON (*n*=50)	*P*	IFG (*n*=12)	*P*	IGT (*n*=15)	*P*	T1D (*n*=25)	*P*	T2D CON (*n*=29)	*P*	DN (*n*=24)	*P*	DR (*n*=34)	*P*	DPN (*n*=50)	*P*
Glu (mmol/L)	*r*1	-0.170	0.239	-0.220	0.491	0.065	0.819	0.137	0.515	-0.061	0.754	-0.023	0.914	0.030	0.866	0.115	0.427
	*r*2	-0.482	<0.001*∗*	-0.004	0.991	-0.079	0.780	-0.389	0.055	-0.262	0.170	-0.279	0.186	-0.207	0.241	-0.159	0.269
	*r*3	-0.063	0.663	0.068	0.834	0.387	0.154	0.332	0.105	0.231	0.228	-0.081	0.706	-0.179	0.310	0.139	0.335
	*r*4	-0.109	0.453	-0.011	0.974	0.335	0.222	-0.171	0.413	0.003	0.986	-0.155	0.468	-0.086	0.629	-0.001	0.994
HbA1c (%)	*r*1	-0.233	0.104	0.095	0.768	0.265	0.340	0.093	0.658	0.258	0.177	0.122	0.570	0.098	0.580	0.095	0.511
	*r*2	-0.409	0.003*∗*	-0.212	0.507	0.032	0.909	0.139	0.507	-0.100	0.606	0.104	0.629	-0.055	0.759	0.175	0.224
	*r*3	-0.060	0.681	0.050	0.877	0.518	0.048*∗*	0.254	0.221	0.091	0.638	0.043	0.843	0.047	0.791	0.180	0.211
	*r*4	-0.089	0.539	-0.007	0.983	0.483	0.068	0.289	0.161	0.138	0.476	0.222	0.298	-0.128	0.469	0.110	0.448
Cr (mg/L)	*r*2	0.419	0.002*∗*	-0.154	0.634	0.464	0.081	0.283	0.170	0.557	0.002*∗*	0.774	<0.001*∗*	0.470	0.006*∗*	0.500	<0.001*∗*
	*r*3	0.096	0.505	0.121	0.707	0.216	0.438	0.390	0.054	0.022	0.910	0.228	0.284	0.143	0.419	0.149	0.302
	*r*4	0.366	0.009*∗*	-0.655	0.021*∗*	0.082	0.771	-0.169	0.421	-0.037	0.849	-0.157	0.463	0.112	0.528	-0.106	0.465
UCr (mg/L)	*r*2	-0.116	0.421	-0.409	0.186	-0.041	0.884	0.096	0.647	-0.153	0.428	0.217	0.308	0.068	0.704	0.127	0.379
	*r*4	0.665	<0.001*∗*	-0.032	0.921	0.623	0.013*∗*	0.186	0.375	0.010	0.957	0.298	0.157	0.374	0.030*∗*	0.255	0.074
Fe (mg/L)	*r*4	-0.020	0.892	-0.102	0.753	-0.243	0.383	0.174	0.406	0.097	0.616	-0.063	0.771	0.021	0.905	0.092	0.525

*r*1 means the correlation coefficient between serum Cr and other indicators; *r*2 is the correlation coefficient between serum Fe and other indicators; *r*3 is the correlation coefficient between urinary Cr and other indicators; *r*4 is the correlation coefficient between urinary Fe and other indicators.*∗P*<0.05 means significant difference.

**Table 3:** Serum parameters in T2D patients treated with simvastatin.

Simvastatin (*n*=24)			
	Pretreatment	Posttreatment	*P*
Cr	0.165(0.087-0.241)	0.178(0.067-0.234)	0.695
UCr	0.048(0.020-0.064)	0.034(0.021-0.057)	0.837
Fe	2.235(1.461-2.742)	2.147(1.685-2.852)	0.680
UFe	0.370(0.173-0.735)	0.320(0.213-0.770)	0.724

Data presentation and abbreviations' spelt-out forms are the same as the description for Table 1.

## Data Availability

The data used to support the findings of this study are available from the corresponding author upon request.
